# Impact of adding hand-washing and water disinfection promotion to oral cholera vaccination on diarrhoea-associated hospitalization in Dhaka, Bangladesh: evidence from a cluster randomized control trial

**DOI:** 10.1093/ije/dyx187

**Published:** 2017-09-02

**Authors:** Nusrat Najnin, Karin Leder, Firdausi Qadri, Andrew Forbes, Leanne Unicomb, Peter J Winch, Pavani K Ram, Elli Leontsini, Fosiul A Nizame, Shaila Arman, Farzana Begum, Shwapon K Biswas, John D Clemens, Mohammad Ali, Alejandro Cravioto, Stephen P Luby

**Affiliations:** dyx187-1 ICDDR,B: International Centre for Diarrhoeal Disease Research, Dhaka, Bangladesh,; dyx187-2 Department of Epidemiology and Preventive Medicine, Monash University, Melbourne, VIC, Australia,; dyx187-3 Johns Hopkins Bloomberg School of Public Health, Baltimore, MD, USA,; dyx187-4 Department of Epidemiology and Environmental Health, University at Buffalo, Buffalo, NY, USA,; dyx187-5 Department of Medicine, Rangpur Medical College Hospital, Rangpur, Bangladesh,; dyx187-6 UCLA Fielding School of Public Health, Los Angeles, CA, USA,; dyx187-7 Global Evaluative Sciences, Inc., Seattle, WA, USA and; dyx187-8 Stanford Woods Institute, Stanford University, Stanford, CA, USA

**Keywords:** Vaccine, hand-washing, water treatment, diarrhoea, hospitalization

## Abstract

**Background:**

Information on the impact of hygiene interventions on severe outcomes is limited. As a pre-specified secondary outcome of a cluster-randomized controlled trial among >400 000 low-income residents in Dhaka, Bangladesh, we examined the impact of cholera vaccination plus a behaviour change intervention on diarrhoea-associated hospitalization.

**Methods:**

Ninety neighbourhood clusters were randomly allocated into three areas: cholera-vaccine-only; vaccine-plus-behaviour-change (promotion of hand-washing with soap plus drinking water chlorination); and control. Study follow-up continued for 2 years after intervention began. We calculated cluster-adjusted diarrhoea-associated hospitalization rates using data we collected from nearby hospitals, and 6-monthly census data of all trial households.

**Results:**

A total of 429 995 people contributed 500 700 person-years of data (average follow-up 1.13 years). Vaccine coverage was 58% at the start of analysis but continued to drop due to population migration. In the vaccine-plus-behaviour-change area, water plus soap was present at 45% of hand-washing stations; 4% of households had detectable chlorine in stored drinking water. Hospitalization rates were similar across the study areas [events/1000 person-years, 95% confidence interval (CI), cholera-vaccine-only: 9.4 (95% CI: 8.3–10.6); vaccine-plus-behaviour-change: 9.6 (95% CI: 8.3–11.1); control: 9.7 (95% CI: 8.3–11.6)]. Cholera cases accounted for 7% of total number of diarrhoea-associated hospitalizations.

**Conclusions:**

Neither cholera vaccination alone nor cholera vaccination combined with behaviour-change intervention efforts measurably reduced diarrhoea-associated hospitalization in this highly mobile population, during a time when cholera accounted for a small fraction of diarrhoea episodes. Affordable community-level interventions that prevent infection from multiple pathogens by reliably separating faeces from the environment, food and water, with minimal behavioural demands on impoverished communities, remain an important area for research.

## Introduction

Diarrhoeal diseases continue to be a major cause of morbidity and mortality in low-income countries, including Bangladesh.^1–4^ In Bangladesh, parents of approximately 36% of the children < 5 years of age, who suffer from diarrhoea, seek care from a hospital or health care centre.^5^

Water, sanitation and hygiene interventions can effectively interrupt transmission of gastrointestinal pathogens to reduce diarrhoea.^6^ The optimum long-term solution in low-income countries would be to build and maintain a water and sanitary infrastructure that consistently separates faecal waste from water and food supplies but, for complex reasons including limited supply, poor governance and low water tariffs leading to lack of funding, achieving this goal in the short term is not feasible.^7^ Therefore, interim approaches for immediate implementation to reduce disease burden would be useful.

One option for preventing diarrhoea is vaccination for specific gastrointestinal pathogens. In cholera-endemic areas, cholera vaccine has been demonstrated to reduce morbidity and mortality from cholera disease including all-cause diarrhoea-associated hospitalization when the burden of cholera was high.^8–12^ Two other rigorously evaluated low-cost approaches to prevent diarrhoeal disease include treatment of water at point of use and promoting hand-washing with soap.^13^^,^^14^ In rural Bangladesh, only 1% of people wash their hands with soap before eating or feeding children and only 14% wash their hands with soap after defecation.^15^ Boiling is the usual method for water treatment in urban areas especially where gas supply is available, but in a study conducted in urban Bangladesh only 37% boiled their water.^16^

Efficacy studies focusing on promoting water treatment at point of use and hand-washing with soap have targeted up to 4000 households in various countries where diarrhoea is a leading cause of death.^13^^,^^14^ However, whether these approaches are effective when implemented on a larger scale is unclear.^17^^,^^18^ Additionally, the efficacy of such interventions has been assessed mainly through potentially biased self-reported diarrhoea episodes rather than using an observable measurement to determine reduction in hospitalization rates for diarrhoea.^19^ It is also unclear whether combining vaccination with behaviour-change interventions incrementally increases health benefits.

In 2011, we conducted a cluster-randomized controlled trial that continued over 2 years among ∼60 000 low-income households of metropolitan Dhaka, Bangladesh.^9^ This current paper reports a pre-specified secondary outcome, namely to examine effects of an intervention to promote hand-washing with soap and also drinking water disinfection in addition to oral cholera vaccination, on diarrhoea-associated hospitalization. We hypothesized that participants in the cholera vaccine-only intervention area would have lower hospitalization rates compared with the control. We also hypothesized that the combination of cholera vaccine plus hand-washing and point of use water treatment would further lower hospitalization rates for diarrhoea, compared with the vaccine-only intervention or control area.

## Methods

### Trial design, context and participant selection

We conducted a cluster-randomized controlled trial in diarrhoea-prone communities of urban Dhaka. Details of the study methods have been published previously (ClinicalTrials.gov Registration number: NCT01339845).^9^ The study areas were divided into 90 geographical clusters, each surrounded by a 30-m buffer zone to limit contamination of the interventions across clusters. Twelve governmental and non-governmental hospitals/clinics with inpatient facilities in and around the study area, and which were accessible to study participants, were included in the study. For the purpose of this study, data were collected from these hospitals/clinics to identify diarrhoea-associated hospitalization of the study participants.

### Randomization

Ninety clusters were randomly assigned into three groups: (i) cholera vaccine alone (denoted as ‘vaccine-only’); (ii) combined cholera vaccine and behaviour-change intervention (denoted as ‘vaccine-plus-behaviour-change’); and (iii) control group which continued standard habits and practices. Blinding of the study investigators and participants was not possible. 

### Intervention

#### Vaccine

**Figure 1:**
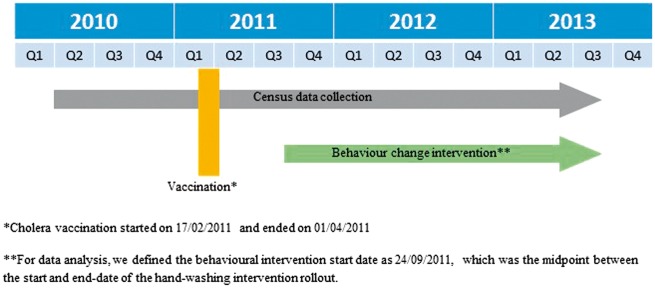
Study timeline.

The WHO pre-qualified the vaccine Shanchol^TM^ (ShanthaBiotechnics) as safe and effective against cholera,^20^^,^^21^ and it was approved for research purposes in this study. Details of vaccine transportation, storage and administration have been reported.^9^ Two vaccine doses were administered at least 14 days apart at no cost to non-pregnant participants aged ≥ 1 year. Vaccination was done between 17 February 2011 and 1 April 2011 (Figure 1).

#### Hand-washing and water treatment behavioural intervention

The hand-washing and water treatment intervention included distribution of enabling hardware and interpersonal counselling aided by support print materials. The behaviour change strategy was guided by the Integrated Behavioural Model for Water Sanitation and Hygiene (IBM-WASH) theoretical framework.^22^^,^^23^ Where households were organized into compounds with several households sharing a common water source, kitchen, and toilets, hardware enabling hand-washing and water treatment was provided at the compound level. The interpersonal counselling targeted people at both compound and household levels.

Dushtha Shasthya Kendra (DSK), a non-governmental organization, delivered the behavioural intervention and hardware. Within 3 months of cholera vaccination, community health promoters visited each compound and rolled out the hand-washing intervention, with the point of use water treatment intervention rolled out 3 months later.

**Figure 2:**
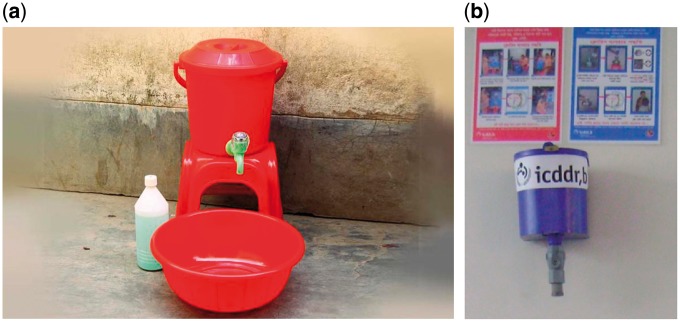
Hand-washing station [includes bucket with tap, bowl, and soapy water (a) and point of use water treatment hardware including chlorine dispenser and instruction sheets (b)].

Hand-washing hardware, provided free of charge, consisted of a bucket with a tap, a bowl where rinse water could accumulate, and a soapy water bottle (Figure 2a). Soapy water was prepared by mixing a commercially available powdered detergent with 1.5 l of water in a plastic bottle with a hole punched in the cap.^24^ Promoters encouraged all households to either purchase inexpensive detergent sachets (∼US$0.03) to make soapy water, or purchase bars of soap (∼US$ 0.35). They encouraged all household members to wash hands regularly, especially after defaecation and before preparing food, and carefully explained all salient benefits. The latter were based on literature review and site-specific formative research, guided by the IBM-WASH theoretical framework.^22^^,^^23^ The water treatment hardware consisted of a chlorine dispenser containing liquid sodium hypochlorite (Figure 2b).^25^ Study participants were encouraged to add chlorine to their own water vessels, which were marked to match the dispensed chlorine dosage with the size of the vessel. Benefits were again explained.

Promoters visited each compound at least three times during each of the first 2 months after placement of each hardware type. After full implementation, the frequency of visits was reduced to twice per month. During visits, along with promoting behavioural interventions, hardware-related problems (breakage/leakage) were addressed.

### Study timeline

For data analysis, we defined the intervention outcome-monitoring start date as 24 September 2011 (Figure 1). We terminated follow-up for all individuals on 31 August 2013 or, if they had died or permanently out-migrated, their final date of assessment; during this monitoring period, study participants in the vaccine-plus-behaviour-change area (including in-migrants) continuously received the behaviour-change interventions.

### Measurements

The pre-specified outcome of interest was the rates of hospital admission for diarrhoea of any clinical severity. We also conducted an exploratory analysis of the impact of the interventions on severe diarrhoea hospitalization. Severe diarrhoea was defined by the presence of at least two of the following signs and symptoms: sunken eyes, dry tongue, thirst, irritability, less active than usual, skin pinch going back slowly, low volume radial pulse along with inability to drink, or absence of radial pulse. The number of diarrhoea-associated hospitalizations (defined as ≥ 3 loose/liquid stools within 24 h^26^) was collected through hospital surveillance. The number of person-years observed was estimated based on information collected through 6-monthly census updates, during which data collectors visited each house in the study areas to obtain information on births, deaths and migrations of individuals.^9^

Each month, a separate survey was conducted among a different set of 200 randomly selected study participants in the vaccine-plus-behaviour-change area, and 100 participants in each of the vaccine-only and control areas, to determine uptake of the hand-washing and water treatment interventions. Unannounced home visits assessed intervention uptake by examining for the presence of soap/soapy water and water in the most convenient place for hand-washing. Presence of residual chlorine in stored drinking water was tested using colorimetre (HACH LANGE GmbH, USA).

### Statistical methods

#### Primary analysis

Using 6-monthly census data, we compared baseline demographic characteristics of study participants across the three intervention areas, and identified individuals who in- or out-migrated into the study area after outcome-monitoring commencement. Since the behavioural interventions were geographically based, people could not take the intervention-enabling hardware with them following migration out of the vaccine-plus-behaviour-change area. Conversely, people migrating into the vaccine-plus-behaviour-change area gained access to interventions. Our analysis accumulated person-years for each individual in a time-dependent manner according to their time at risk in each trial area. Specifically, when a person moved from one trial area to a different trial area, or migrated for the first time into the overall study area, we waited 14 days before beginning to allocate their person-time to the in-migrated trial area so that the effect of their previous exposures could be reduced and their new exposure established. Once a person migrated out of the overall study area altogether, we stopped accumulating his/her person-time. We allowed multiple hospitalizations per individual by continuing accumulation of person-years after hospitalization.

We calculated the diarrhoea-associated hospitalization incidence by counting the number of admissions from each study area during the outcome-monitoring period, and summed the person-time that study participants contributed to each trial area. We adjusted hospitalization incidence rates for the cluster-randomized trial design, and the potential multiple hospitalizations per individual using robust standard errors applied at the cluster level. To calculate the hazard ratio for diarrhoea-associated hospitalization of any severity, we compared incidence of hospitalization for diarrhoea in the vaccine-plus-behaviour-change area with the control and to the vaccine-only areas using Cox proportional hazards regression with cluster-robust standard errors. Results were adjusted for age, sex, education and pre-intervention individual-level hospitalizations.

We divided the 2-year outcome-monitoring period into quartiles (term 1 to term 4) to examine the consistency of the intervention effect on incidence of hospitalization over time, using intervention*quartile interaction terms in the Cox proportional hazards regression models. We assessed effect modification of the intervention by age in a similar manner with interaction terms. 

#### Supplementary analyses

These included:

- an analysis restricted to individuals who resided in the study area at the outcome-monitoring start date and remained in their original intervention area for the entire study duration; this analysis excluded new in-migrations after the outcome-monitoring start date;
- an analysis allocating all person-time to the trial area of each individual at the outcome-monitoring start date, regardless of later migrations to other areas, and excluding in-migration after the outcome-monitoring start date.

Details regarding sample size calculations for the primary study outcome have been published elsewhere.^9^

### Ethics

Informed consent from an adult study participant was obtained from each household. The study protocol was reviewed by Human Subject Committee at icddr,b, and the International Vaccine Institute.

## Results

### Participant characteristics and migration

During the 6–12 months before the outcome-monitoring started, 314 748 people lived in the study area (Table 1). Demographic characteristics were similar across the three areas except educational status, self-reported drinking water treatment practices, and presence of sanitary latrines which were slightly higher in the vaccine-plus-behaviour-change area (Table 1).

**Table 1.** Demographic characteristics across the intervention areas before outcome-monitoring started^a,b^

Demographics	Vaccine-only area (*n* = 109700) %	Vaccine-plus-behaviour-change area (*n* = 107134) %	Control area (*n* = 97914) %
Age (mean, SD)	23.3 (15.6)	23.4 (15.5)	23.4 (15.7)
≤5years	13.1	13.2	13.3
>5–15 years	19.6	19.2	19.9
>15–50 years	61.9	62.2	61.1
>50 years	5.5	5.5	5.7
Sex (male)	48.2	48.7	48.7
Educational status			
No formal education (includes children < 5years)	43.8	41.4	43.9
Below primary	17.4	17.5	17.6
Primary and some secondary	30.8	31.7	30.0
Above secondary	8.0	9.4	8.5
Number of people in a family (median, interquartile range)	5 (2)	5 (2)	5 (2)
Number of months living in this house (median, interquartile range)	12 (57)	12 (57)	12 (56)


Characteristics of households	Vaccine area (*n* = 27341) %	Vaccine-plus-behaviour-change area (*n* = 26794) %	Control area (*n* = 24393) %
Source of drinking water (WASA supply water)^c^	99.9	99.7	99.9
Treat drinking water (yes)	52.6	58.7	54.6
Boil water	51.5	56.4	53.1
Filter water	0.7	1.2	0.9
Chemical treatment	0.4	1.1	0.6
Distance from source of drinking water to the kitchen in centimeters (median, interquartile range)	457 (457)	457 (457)	457 (457)
Shared kitchen (yes)	89.6	93.0	87.6
Shared toilet (yes)	96.7	96.0	95.8
Type of toilet (direct observation)			
Sanitary latrine with or without flush	70.5	81.3	78.5
Non-sanitary	28.5	17.9	21.3
Use open space	1.0	0.8	0.2
Waste disposal (fixed place)	81.8	84.7	79.3
House construction material			
Roof			
Corrugated iron	87.1	84.5	83.2
Brick/concrete	12.8	15.4	16.7
Bamboo/wood/other	0.1	0.1	0.1
Floor			
Brick/concrete	90.3	90.4	91.5
Bamboo/wood/other	9.7	9.6	8.5
Wall			
Corrugated iron	28.2	23.9	26.0
Brick/concrete	68.4	73.9	70.1
Bamboo/wood/other	3.4	2.2	3.9
Number of rooms in the house (mean, SD)	1.1 (0.4)	1.2 (0.5)	1.2 (0.5)
Monthly rent paid (median, interquartile range) (US$)^d^	25.8 (12.9)	25.8 (12.9)	25.8 (12.2)
Monthly household expenditure (median, interquartile range) (US$)	103.0 (51.3)	105.6 (52.2)	104.3 (49.6)
Monthly average savings (median, interquartile range) (US$)	0 (3.8)	0 (2.6)	0 (3.1)


WASA, Water and Sewerage Authority; BDT, Bangladeshi Taka.

^a^Unique person identification (ID); some categories do not sum to 100% because of rounding.

^b^Pre-intervention period data were used in this table to: (i) avoid migration issues that occurred during intervention period and possibly could have changed the demographics across the intervention/control areas; and (ii) to assess pre-intervention period drinking water treatment and hygiene status.

^c^Other sources of drinking water include well, bottled water, water vendor and pond/canal/river.

^d^1 USD = 77.7 BDT (average exchange rate during 2012).

**Figure 3:**
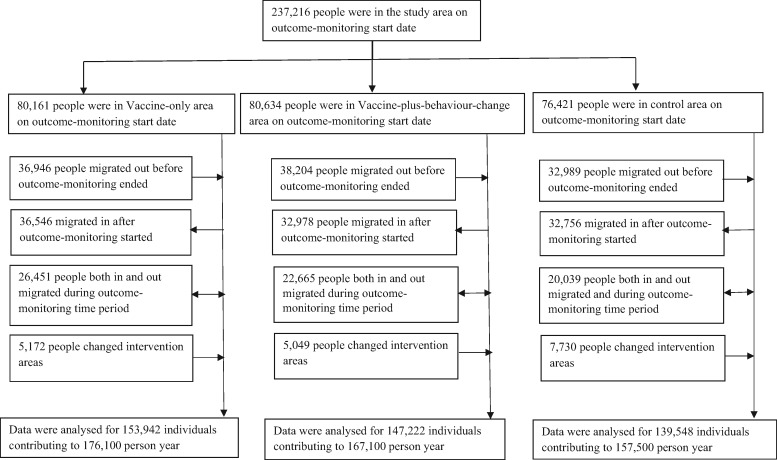
Participant flow during the study outcome-monitoring time period.

We identified 429 995 people who were in the study area at some time point during the outcome-monitoring period and contributed to 500 700 person-years of data; of them, 177 299 people left the study area before outcome-monitoring ended (Figure 3). The median duration of residence in the same house was 12 months. During intervention period, ∼4% people (*n* = 17 951) changed areas, but despite migration, the three areas remained balanced by demographic characteristics (data not shown).

### Intervention uptake

Two-dose vaccine coverage during mass immunization was ∼65%,^9^ but dropped to ∼58% 6 months later, at the start of our analysis, due to population migration. Data from 24-monthly surveys collected from a subset of 7542 households showed that soap/soapy water and water was present at 45% (1729/3886) households of the primary hand-washing stations of the vaccine-plus-behaviour-change area, 22% (438/1965) of the vaccine-only and 28% (556/1991) of the control area. Residual chlorine, indicating uptake of the chlorine dispenser, was present in the stored drinking water of 4% (160/3886) of households in the vaccine-plus-behaviour-change area and none in the other two areas.

Presence of indicators for both hand-washing and point of use water treatment interventions were ∼4% higher among people who stayed in the study area for at least 1 year after the intervention started, compared with those who migrated in or out or both.

### Diarrhoea-associated hospitalization rates

During the outcome-monitoring period, the overall diarrhoea hospitalization rate for the primary analysis was 9.6/1000 person-years (95% CI: 8.8–10.4). The hospitalization rate was comparatively similar across the areas (vaccine-only 9.4/1000 person-years; vaccine-plus-behaviour-change 9.6/1000 person-years; control 9.7/1000 person-years) (Table 2). The results remained similar after considering people migrating from vaccine-only/vaccine-plus-behaviour-change areas to control areas as remaining vaccinated (Supplementary Table 1, available as Supplementary data at *IJE* online). The hospitalization rate was also relatively similar across the different areas over terms 1 to 4 (interaction between areas and terms: *P* = 0.67) (Table 3). No interaction was present between areas and age (data not shown). During the period, 47% (*n* = 2270) of diarrhoea-associated hospitalizations were due to severe diarrhoea. Although the severe-diarrhoea-associated hospitalization rates were slightly lower in the vaccine-plus-behaviour-change area, the 95% CIs overlapped each other [severe diarrhoea hospitalization rate: vaccine-only 4.7/1000 person-years (95% CI: 4.1–5.6); vaccine-plus-behaviour-change 4.1/1000 person-years (95% CI: 3.4–5.0); control 4.7/1000 person-years (95% CI: 3.9–5.8)].

**Table 2.** Hospitalization rates and person-years during outcome-monitoring period by treatment areas (cluster-adjusted)^a^

Study areas	Number of people	Number of person-years (1000)	Number of hospitalizations	Hospitalizations/1000 person-years (95% CI)	Hazard ratio (95% CI)	*P*-value*
Control	145821	164.0	1600	9.7 (8.3–11.6)	1.0	–
Vaccine-only	149839	169.6	1586	9.4 (8.3–10.6)	0.96 (0.78–1.17)	0.69
Vaccine-plus-behaviour-change	147222	167.1	1596	9.6 (8.3–11.1)	0.98 (0.79–1.22)	0.85

^a^Primary analysis

*P-value for comparison with control.

**Table 3.** Diarrhoea-associated hospitalization rates^a^ and hazard ratios^b^ before and during outcome-monitoring period by intervention and control areas (primary analysis)

Study areas	Hospitalizations/1000 person-years Before outcome-monitoring start date		Outcome-monitoring period							
	6–12 months before outcome-monitoring started 24/09/2010 to 23/03/2011	0–6 months before outcome-monitoring started^c^ 24/03/2011 to 23/09/2011	Term 1 24/09/2011 to 23/03/2012		Term 2 24/03/2012 to 23/09/2012		Term 3 24/09/2012 to 23/03/2013		Term 4 24/03/2012 to 31/08/2013	
			Hospitalizations/1000 person- years	Hazard ratio 95% CI	Hospitalizations/1000 person- years	Hazard ratio 95% CI	Hospitalizations/1000 person- years	Hazard ratio 95% CI	Hospitalizations/1000 person- years	Hazard ratio 95% CI
All study areas combined	2.4	12.7	9.6	–	10.2	–	7.6	–	11.0	–
Control	1.2	13.7	9.8	1.0	10.3	1.0	7.9	1.0	11.2	1.0
Vaccine-only	3.2	12.5	8.8	0.90 (0.7–1.2)	10.1	0.98 (0.8–1.2)	7.8	0.99 (0.7–1.4)	10.9	0.97 (0.7–1.4)
Vaccine-plus-behaviour-change	2.8	12.1	10.2	1.04 (0.8–1.4)	10.4	1.01 (0.8–1.2)	7.1	0.90 (0.6–1.3)	10.7	0.95 (0.7–1.3)

^a^Results are cluster-adjusted.

^b^Results are only cluster-adjusted. Results that are adjusted for age, sex, education, toilet type, pre-intervention period hospitalizations and cluster were almost similar to the unadjusted results (data not shown).

^c^Cholera vaccine was delivered during this period.

**Figure 4:**
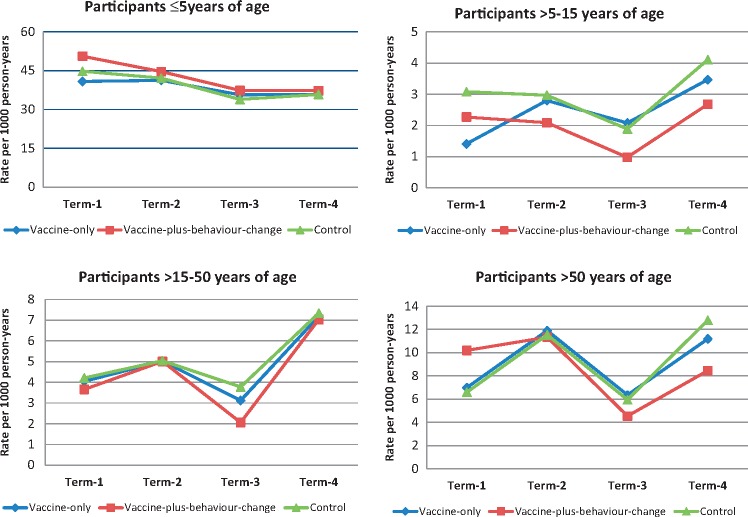
Hospitalization rates for different age groups across the intervention areas* during outcome-monitoring period**. *The *P*-value of interaction between areas, time and age was 0.12. **Term 1: 24 September 2011 to 23 March 2012; term 2: 24 March 2012 to 23 September 2012; term 3: 24 September 2012 to 23 March 2013; term 4: 24 March 2013 to 31 August 2013.

Hospitalization rates were higher among children aged ≤ 5 years compared with the other age groups (Figure 4). The *P-*value for three-way interaction between intervention/control areas, intervention period and age was 0.12, indicating no rate differences by age over time beyond that expected by chance. The hospitalization rates among study participants ≥ 1 year of age (excluding children < 1 year from the time of vaccination and onwards) were similar across the study areas during the outcome-monitoring period (Supplementary Table 2, available as Supplementary data at *IJE* online).

The hospitalization rate among the subgroup of people who remained in the same location for the entire intervention period was slightly lower in the vaccine-plus-behaviour-change area compared with other areas, but the 95% CIs overlapped each other—hospitalization rate: vaccine-only 9.4/1000 person-years (95% CI: 8.3–10.7); vaccine-plus-behaviour-change 9.0/1000 person-years (95% CI: 7.6–10.6); control 9.7/1000 person-years (95% CI: 8.3–11.5). The absolute and relative rates of hospitalization using the supplementary analysis (ii) were only negligibly different from the primary analysis (Supplementary Table 3, available as Supplementary data at *IJE* online).

## Discussion

This study reports an observable measure of the impact of combined hand-washing and point of use water treatment intervention plus cholera vaccination on hospitalization for diarrhoeal disease, examined through a large-scale, community-based intervention trial. Despite using an effective cholera vaccine and culturally adapted behaviour-change interventions, we found no significant impact of combined vaccine-plus-behaviour-change intervention on rates of hospitalization with diarrhoea or hospitalization with severe diarrhoea.

In an earlier study, cholera vaccine reduced all-cause severely dehydrating diarrhoea-associated hospitalization.^8^ In the current study, in an earlier analysis vaccination reduced the incidence of diarrhoea attributable to *V. cholerae*,^9^ yet we did not observe any significant impact of cholera vaccine alone on all-cause diarrhoea hospitalization, presumably because the cholera incidence was too low during the study period to make a detectable contribution to overall hospitalization rates for all-cause diarrhoea. Indeed, the culture-confirmed cholera cases accounted for ∼7% of total number of cases of diarrhoea-related hospitalization, well below the years immediately preceding the study.^9^ In countries like Bangladesh where cholera is endemic, the magnitude of cholera incidence can vary from year to year.^27^ Additionally, the high migration rate diluted cholera vaccination coverage of the intervention areas, thus reducing the impact of vaccine on diarrhoea-associated hospitalization.

The vaccine-plus-behaviour-change area received intervention hardware and instructions to wash their hands and treat their drinking water, in addition to receiving cholera vaccine. The corresponding behaviour-change strategy was tested in a pilot study to estimate acceptability before roll-out in the main trial.^22^ Chlorinating water and hand-washing promotion have been effective in reducing self-reported diarrhoeal diseases in small-scale efficacy studies.^13^^,^^14^ However, we observed no statistically significant overall or age-specific impact on hospitalization outcomes.

One reason for the lack of impact of the behavioural intervention may have been because of the low uptake. We ideally would have examined diarrhoea hospitalization rates among those who had good intervention uptake versus those who did not, but we could not link the intervention uptake data that was collected from only a small sub-sample of the study population to the hospitalization data. Identifying and reporting details of the reasons for poor uptake of these previously tested interventions will be assessed and reported separately, but may be related to difficulty of delivering the behaviour change intervention with high quality on a large scale.^17^^,^^18^

Our indicator of hand-washing behaviour uptake was the presence of soap and water at the primary hand-washing station. Among the vaccine-plus-behaviour-change population, the hand-washing indicator was only 17% points (45% vs 28%) higher than in the control area. Even though this is a commonly used indicator to assess hand-washing uptake,^15^^,^^28^ it does not ensure that people actually wash their hands or use soap. Based on the presence of residual chlorine in drinking water, only 4% people used the chlorine dispenser. This was disappointing but not entirely unexpected, as the pilot study had also shown low uptake and hardware-related problems which were unresolved when the vaccine became available and main trial commenced. Low uptake of chlorine-based water treatment products has been reported in similar contexts.^17^^,^^29^ For example, a study conducted in urban Dhaka in 2009, promoting chlorine-based products detected residual chlorine in only ∼8% of households.^29^ The taste and smell of chlorine-treated water is a commonly reported barrier.^30^ Moreover, a large number of the study participants migrated out of the study area before completion of the 2-year follow-up, thereby limiting the consistency of participants’ exposure to the intervention. However our analysis, restricted to people who stayed in the study area for the entire study period, also showed no reduction in diarrhoea hospitalization, despite a slightly higher uptake of interventions compared with those who migrated. The hospitalization rate was comparatively lower during the 6–12 months preceding theintervention period. The reason for this is unknown, but it could be due to variations in diarrhoea rate at the community level over time or to delays before the surveillance was fully capturing all cases.

In conclusion, we observed limited public health impact, by the combination of oral cholera vaccine and behavioural interventions to improve drinking water quality and hand-washing behaviour, on the rate of hospitalized diarrhoea in the setting under study. Developing better behavioural interventions that increase water treatment and hand-washing remain important in areas where marginal improvement is possible. Whereas the low rate of cholera and high rate of population migration account for the limited impact of oral cholera vaccination, the failure of the drinking water and hand-washing intervention underscores the need for investment in research to improve the pace and effectiveness of community-wide interventions that separate human faeces from the environment, food and water supply of low-income country residents.

## Supplementary Data

Supplementary data are available at *IJE* online. 

## Funding

This work was supported by the Bill & Melinda Gates Foundation [Grant Number: OPP50419]. icddr,b acknowledges with gratitude the commitment of the Bill and Melinda Gates Foundation to its research efforts. The funding agency provided input on study design but had no role in data collection, analysis or manuscript preparation. The centre is also thankful to the governments of Bangladesh, Canada, Sweden and the UK for providing core/unrestricted support.

## Supplementary Material

Supplementary Appendix

## References

[1] Liu L, Oza S, Hogan D Global, regional, and national causes of child mortality in 2000–13, with projections to inform post-2015 priorities: an updated systematic analysis. Lancet 2015;385**:** 430–40. 25280870 10.1016/S0140-6736(14)61698-6

[2] Morris JG Cholera—modern pandemic disease of ancient lineage. Emerg Infect Dis 2011;17**:** 2099–104. 22099113 10.3201/eid1711.111109 PMC3310593

[3] Walker CLF, Perin J, Aryee MJ, Boschi-Pinto C, Black RE Diarrhea incidence in low-and middle-income countries in 1990 and 2010: a systematic review. BMC Public Health 2012;12**:** 220. 22436130 10.1186/1471-2458-12-220 PMC3323412

[4] Black RE, Cousens S, Johnson HL Global, regional, and national causes of child mortality in 2008: a systematic analysis. Lancet 2010;375**:** 1969–87. 20466419 10.1016/S0140-6736(10)60549-1

[5] National Institute of Population Research and Training (NIPORT), Mitra and Associates, and ICF International. Bangladesh Demographic and Health Survey 2014: Key Indicators. Dhaka, Bangladesh, and Rockville, MD: NIPORT, Mitra and Associates, and ICF International, 2015.

[6] Fewtrell L, Kaufmann RB, Kay D, Enanoria W, Haller L, Colford JM Jr Water, sanitation, and hygiene interventions to reduce diarrhoea in less developed countries: a systematic review and meta-analysis. Lancet Infect Dis 2005;5**:** 42–52. 15620560 10.1016/S1473-3099(04)01253-8

[7] McIntosh AC Asian Water Supplies: Reaching the Urban Poor: a Guide and Sourcebook on Urban Water Supplies in Asia for Governments, Utilities, Consultants, Development Agencies, and Nongovernment Organizations. Mandaluyong, Philippines: Asian Development Bank, 2003.

[8] Clemens J, Harris J, Khan M Impact of B subunit killed whole-cell and killed whole-cell-only oral vaccines against cholera upon treated diarrhoeal illness and mortality in an area endemic for cholera. Lancet 1988;331**:** 1375–79. 10.1016/s0140-6736(88)92189-7 2898052

[9] Qadri F, Ali M, Chowdhury F Feasibility and effectiveness of oral cholera vaccine in an urban endemic setting in Bangladesh: a cluster randomized open-label trial. Lancet 2015;386**:** 1362–71. 26164097 10.1016/S0140-6736(15)61140-0

[10] Clemens JD, Sack DA, Harris JR Field trial of oral cholera vaccines in Bangladesh: results from three-year follow-up. Lancet 1990;335**:** 270–73. 1967730 10.1016/0140-6736(90)90080-o

[11] Ali M, Emch M, von Seidlein L Herd immunity conferred by killed oral cholera vaccines in Bangladesh: a reanalysis. Lancet 2005;366**:** 44–49. 15993232 10.1016/S0140-6736(05)66550-6

[12] Longini IM Jr, Nizam A, Ali M, Yunus M, Shenvi N, Clemens JD Controlling endemic cholera with oral vaccines. PLoS Med 2007;4**:** e336. 18044983 10.1371/journal.pmed.0040336 PMC2082648

[13] Clasen T, Schmidt W-P, Rabie T, Roberts I, Cairncross S Interventions to improve water quality for preventing diarrhoea: systematic review and meta-analysis. BMJ 2007;334**:** 782. 17353208 10.1136/bmj.39118.489931.BE PMC1851994

[14] Ejemot-Nwadiaro RI, Ehiri JE, Meremikwu MM, Critchley JA Hand washing for preventing diarrhoea. Cochrane Database Syst Rev 2015;9**:** CD004265. 10.1002/14651858.CD004265.pub3 PMC4563982 26346329

[15] Halder AK, Tronchet C, Akhter S, Bhuiya A, Johnston R, Luby SP Observed hand cleanliness and other measures of hand-washing behavior in rural Bangladesh. BMC Public Health 2010;10**:** 545. 20828412 10.1186/1471-2458-10-545 PMC2944374

[16] Najnin N, Arman S, Abedin J Explaining low rates of sustained use of siphon water filter: evidence from follow-up of a randomized controlled trial in Bangladesh. Trop Med Int Health 2015;20**:** 471–83. 25495859 10.1111/tmi.12448

[17] Luby SP, Mendoza C, Keswick BH, Chiller TM, Hoekstra RM Difficulties in bringing point of use water treatment to scale in rural Guatemala. Am J Trop Med Hyg 2008;78**:** 382–87. 18337330

[18] Olembo L, Kaona FA, Tuba M, Burnham G Safe water systems: An evaluation of the Zambia Clorin program. Johns Hopkins University Mimeograph, 2004.

[19] Schmidt W-P, Cairncross S Household water treatment in poor populations: is there enough evidence for scaling up now? Environ Sci Technol 2009;43**:** 986–92. 19320147 10.1021/es802232w

[20] WHO. Cholera Vaccines. WHO position paper. Geneva: World Health Organization, 2010.

[21] Sur D, Kanungo S, Sah B Efficacy of a low-cost, inactivated whole-cell oral cholera vaccine: results from 3 years of follow-up of a randomized, controlled trial. PLoS Negl Trop Dis 2011;5**:** e1289. 22028938 10.1371/journal.pntd.0001289 PMC3196468

[22] Hulland KR, Leontsini E, Dreibelbis R Designing a handwashing station for infrastructure-restricted communities in Bangladesh using the integrated behavioural model for water, sanitation and hygiene interventions (IBM-WASH). BMC Public Health 2013;13**:** 877. 24060247 10.1186/1471-2458-13-877 PMC3852554

[23] Dreibelbis R, Winch PJ, Leontsini E The integrated behavioural model for water, sanitation, and hygiene: a systematic review of behavioural models and a framework for designing and evaluating behaviour change interventions in infrastructure-restricted settings. BMC Public Health 2013;13**:** 1015. 24160869 10.1186/1471-2458-13-1015 PMC4231350

[24] Amin N, Pickering AJ, Ram PK Microbiological evaluation of the efficacy of soapy water to clean hands: a randomized, non-inferiority field trial. Am J Trop Med Hyg 2014;91**:** 415–23. 24914003 10.4269/ajtmh.13-0475 PMC4125272

[25] Kremer M, Miguel E, Null C, Zwane AP. Sustainability of long-term take-up at point-of-collection chlorine dispensers provided free of charge in rural Western Kenya. *Proceedings of the Water Environment Federation* 2011;**2011:**249–50.

[26] World Health Organization. The Treatment of Diarrhoea: A Manual for Physicians and Other Senior Health Workers. Geneva: WHO, 2005.

[27] Emch M, Feldacker C, Islam MS, Ali M Seasonality of cholera from 1974 to 2005: a review of global patterns. Int J Health Geog 2008;7**:** 1–13. 10.1186/1476-072X-7-31 PMC2467415 18570659

[28] Luby SP, Halder AK, Tronchet C, Akhter S, Bhuiya A, Johnston RB Household characteristics associated with handwashing with soap in rural Bangladesh. Am J Trop Med Hyg 2009;81**:** 882–87. 19861626 10.4269/ajtmh.2009.09-0031

[29] Luoto J, Najnin N, Mahmud M What point of use water treatment products do consumers use? Evidence from a randomized controlled trial among the urban poor in Bangladesh. PloS One 2011;6**:** e26132. 22028817 10.1371/journal.pone.0026132 PMC3197608

[30] Ram PK, Kelsey E, Rasoatiana RRM, Rakotomalala O, Dunston C, Quick RE Bringing safe water to remote populations: an evaluation of a portable point of use intervention in rural Madagascar. Am J Public Health 2007;97**:** 398. 17267727 10.2105/AJPH.2005.073460 PMC1805013

