# Supplementary material for: Impact of adding hand-washing and water disinfection promotion to oral cholera vaccination on diarrhoea-associated hospitalization in Dhaka, Bangladesh: evidence from a cluster randomized control trial
Source: Int J Epidemiol. 2017 Sep 2;46(6):2056–66. doi: 10.1093/ije/dyx187 (PMC5837384; doi:10.1093/ije/dyx187)
Supplement: Supplementary Appendix [file ije-2017-02-0216-file008_dyx187.docx]

Appendix:

Supplementary-table-1: Hospitalisation rates and person years during outcome-monitoring period by treatment areas (cluster-adjusted)*

| Study areas | No. of people | No. of person years (1000) | No. of hospitalization | Hospitalizations/  1000 person-years  (95% CI) | Hazard ratio  (95% CI) | P-value** |
| --- | --- | --- | --- | --- | --- | --- |
| Control | 139,584 | 157.5 | 1531 | 9.7 (8.3, 11.5) | 1.0 | - |
| Vaccine-only | 153,942 | 176.1 | 1655 | 9.4 (8.3, 10.6) | 0.97 (0.79, 1.17) | 0.74 |
| Vaccine-plus-behaviour-change | 147,222 | 167.1 | 1596 | 9.6 (8.3, 11.1) | 0.98 (0.79, 1.22) | 0.88 |

*analysis considering people migrating from vaccine-only/vaccine-plus-behaviour-change areas to control area remained vaccinated and were considered in the vaccine-only area during the analysis

**P-value for comparison with Control area

Supplementary-table-2: Hospitalisation rates among study participants ≥1 year of age during outcome-monitoring period by treatment areas (cluster-adjusted)*

| Study areas | Hospitalizations/  1000 person-years  (95% CI) | Hazard ratio  (95% CI) | P-value** |
| --- | --- | --- | --- |
| Control | 5.5 (4.5, 6.7) | 1.0 | - |
| Vaccine-only | 5.1 (4.3, 6.1) | 0.94 (0.73, 1.21) | 0.63 |
| Vaccine-plus-behaviour-change | 4.8 (3.9, 5.9) | 0.88 (0.67, 1.15) | 0.35 |

* Analysis restricted among people who were ≥1 year of age at the time of vaccination and onwards

**P-value for comparison with Control area

Supplementary-table-3: Overall hospitalisation rates and person years by study area using supplementary analysis (ii)* (cluster adjusted)

| Study area | No. of people | No. of person years (1000) | No. of hospitalization | Hospitalizations/  1000 person-years  (95% CI) | Hazard ratio  (95% CI) | P-value |
| --- | --- | --- | --- | --- | --- | --- |
| Control | 82,538 | 107.3 | 933 | 8.7 (7.4, 10.3) | 1.0 | - |
| Vaccine-only | 87,794 | 112.5 | 897 | 8.0 (7.0, 9.1) | 0.92 (0.75, 1.13) | 0.42 |
| Vaccine-plus-behaviour-change | 89,124 | 111.5 | 930 | 8.3 (7.1, 9.8) | 0.96 (0.78, 1.20) | 0.71 |

*Allocating all person time to the trial area of each individual at the intervention start date, regardless of later migrations to other areas, and excluding in-migration after the intervention start date
